# Splenic Laceration: A Rare Complication of Colonoscopy

**DOI:** 10.7759/cureus.24660

**Published:** 2022-05-02

**Authors:** Shujaa Faryad, Mirza S Ali, Habiba Hussain, Subramanyam Chittivelu

**Affiliations:** 1 Pulmonary and Critical Care Medicine, University of Illinois College of Medicine at Peoria, Peoria, USA

**Keywords:** splenic rupture, splenic hematoma, colonoscopy, splenic laceration, splenic injury

## Abstract

Splenic injury is usually seen with penetrating or blunt abdominal trauma. It is also one of the rare complications of colonoscopy. Various patient and procedural factors have been reported to increase the risk of this dreaded complication. We present a case of splenic injury after outpatient colonoscopy where intra-abdominal adhesions from previous abdominal surgeries were presumed to be the cause of splenic injury. Our patient had improved outcomes with timely diagnosis and intervention.

## Introduction

Colonoscopy is being widely used as a diagnostic and therapeutic tool. It has become a routine procedure. As with any procedure, there are complications associated with colonoscopy too. Splenic injury is one of the rare complications of colonoscopy [1]. Pneumothorax, pneumoperitoneum, colonic volvulus, ileus, mesenteric tears, and vasovagal problems are the other rare complications [2]. Excessive tension on the splenocolic ligament and/or on preexisting adhesions while passing the endoscope through the splenic flexure causing a parenchymal tear or avulsion is reported to be the most common mechanism of injury [3]. Physicians and gastroenterologists should be aware of this complication to decrease morbidity and mortality.

## Case presentation

**Figure 1:**
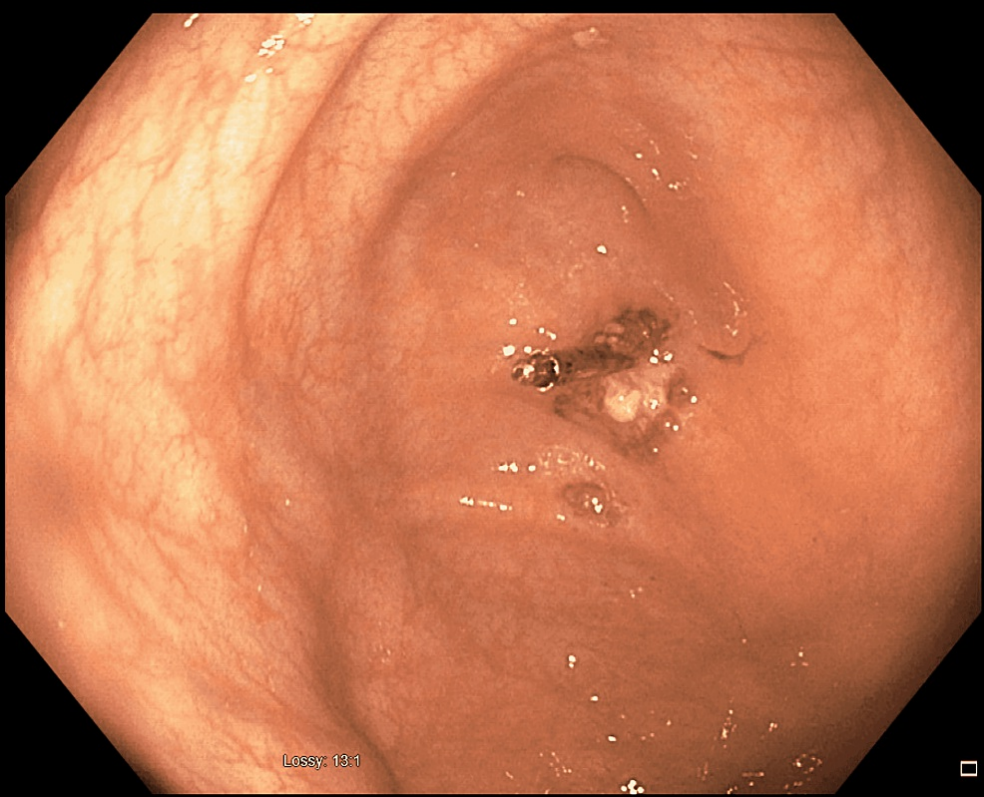
Colonoscopy showing hemostatic clip after cold snare polypectomy.

A 49-year-old Caucasian female presented for an outpatient esophagogastroduodenoscopy (EGD) and Colonoscopy for evaluation of nausea, vomiting, and chronic constipation. She had a history of irritable bowel syndrome constipation-predominant, laparoscopic cholecystectomy, hysterectomy, and paroxysmal supraventricular tachycardia (SVT). Both procedures were performed without difficulty. One cecal polyp was removed with the application of a hemostatic clip (Figure 1).

**Figure 2:**
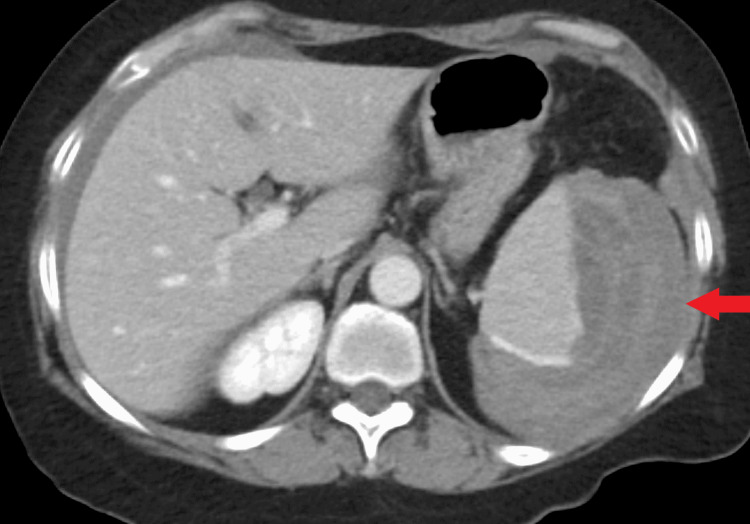
Axial CT abdomen with contrast showing subcapsular perisplenic hematoma (red arrow).

**Figure 3:**
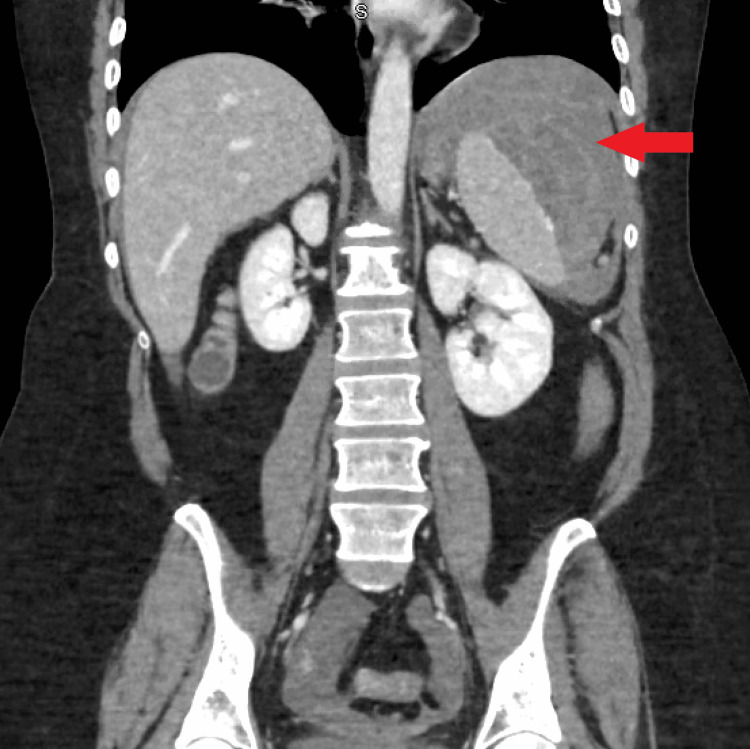
Coronal CT abdomen with contrast showing subcapsular perisplenic hematoma (red arrow).

The EGD was unremarkable. While in the post-anesthesia care unit, the patient developed acute left upper quadrant abdominal and flank pain. Vital signs were normal. Physical examination revealed tenderness in the left upper quadrant, without distention or rigidity. Blood work showed a drop in hemoglobin (Hgb) and hematocrit from 14.7 to 9.7 (N:12-15 g/dl) and 45 to 29.0 (N:36-47%), respectively. X-ray kidney, ureter, and bladder (KUB) was unremarkable. CT abdomen & pelvis with contrast revealed 12.3 x 5.7 cm subcapsular peri splenic hematoma with active extravasation (Figures 2, 3).

The patient underwent emergent celiac and splenic artery angiography with coil embolization of the proximal splenic artery. The postoperative course was uneventful. The patient was monitored in the medical ICU and managed with bed rest, analgesia, two units of packed red blood cells, and intravenous fluid hydration. She was discharged home on the fourth post-operative day.

## Discussion

Colonoscopy is considered a safe procedure. Hemorrhage (1% to 2%) and colonic perforation are common complications with an incidence of 1% to 2% and 0.1 to 2% respectively [4]. Splenic injury is a rare but serious complication of colonoscopy with a mortality rate of 5% [5]. Incidence is reported to be around 1/100,000 procedures [6]. It is seen more commonly in females with a mean age of 63 years [6]. The exact mechanism of splenic injury during colonoscopy remains unknown. Excessive traction on the splenocolic ligament and splenocolic adhesions from previous abdominal surgery, certain maneuvers including hooking the splenic flexure to straighten the left colon, and direct trauma to the spleen while passing the scope through the splenic flexure have all been hypothesized as potential causes [7]. Difficult colonoscopy, splenomegaly, biopsies, and polypectomy are also among the known risk factors [7]. Patients usually develop symptoms within 24 hours after the procedure [8]. Left-upper quadrant or generalized abdominal pain is the most common presenting complaint followed by left shoulder pain [9]. Hemodynamic instability can also be seen in the case of significant intraperitoneal bleeding. Contrast-enhanced CT abdomen is the diagnostic modality of choice in suspected cases with a sensitivity of 98.5% [5]. Management depends on the type of injury and hemodynamic status of the patient. Conservative management with observation, serial abdominal exams, and frequent hemoglobin checks is suggested for hemodynamically stable patients. The failure rate of conservative management has been reported up to 44% [10]. For unstable patients or patients who become unstable during conservative management, surgical exploration and angiographic embolization are the potential options. Good colonoscopy technique and left lateral positioning of the patient may reduce the risk of splenic injury by decreasing the effect of gravity and traction. [3] The etiology of splenic injury in our patient remains unclear although suspected intra-abdominal adhesions from previous surgeries might have played a role. She presented within 24 hours of the index procedure and a CT abdomen revealed the splenic injury like most of the reported cases. The angiographic intervention led her to full recovery without the need for more aggressive surgical exploration.

## Conclusions

There has been an increase in the cases of splenic injury with an increasing number of diagnostic and therapeutic colonoscopies. Physicians should have a very low threshold to obtain a contrast-enhanced CT scan of the abdomen in suspected cases of splenic injury. Awareness of this dreaded complication is the key to timely diagnosis and intervention.
